# Supplementary figures and images for: Potential application values of a marine red yeast, Rhodosporidiums sphaerocarpum YLY01, in aquaculture and tail water treatment assessed by the removal of ammonia nitrogen, the inhibition to Vibrio spp., and nutrient composition
Source: PLoS One. 2021 Feb 16;16(2):e0246841. doi: 10.1371/journal.pone.0246841 (PMC7886173; doi:10.1371/journal.pone.0246841)

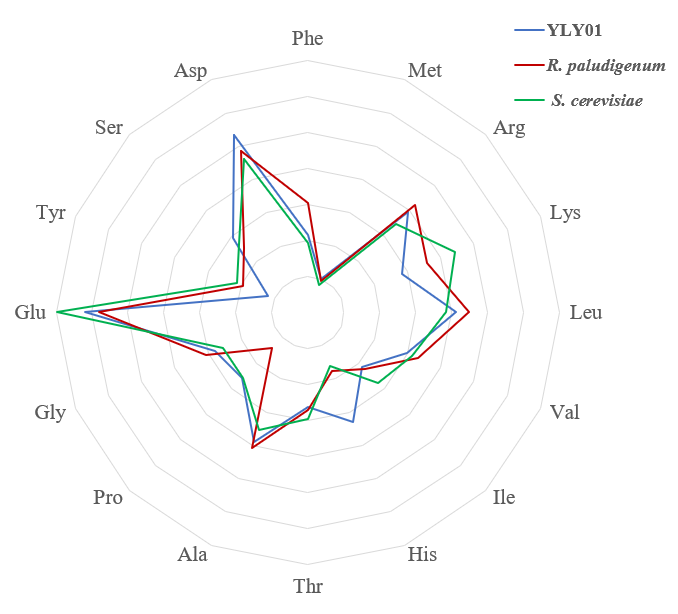


Fig. S1 The radar plot of amino acid composition of three yeasts

Supplement: S1 Fig — (DOCX) [file pone.0246841.s001.docx]
