# Supplementary material for: Potential application values of a marine red yeast, Rhodosporidiums sphaerocarpum YLY01, in aquaculture and tail water treatment assessed by the removal of ammonia nitrogen, the inhibition to Vibrio spp., and nutrient composition
Source: PLoS One. 2021 Feb 16;16(2):e0246841. doi: 10.1371/journal.pone.0246841 (PMC7886173; doi:10.1371/journal.pone.0246841)
Supplement: Attachment — Submitted filename: Review comment PONE-D-20-38047.docx [file pone.0246841.s002.docx]

**Manuscript Review (PONE-D-20-38047) -** Potential application values of a marine red yeast, *Rhodosporidiums sphaerocarpum* YLY01, in aquaculture and wastewater treatment assessed by the removal of ammonia nitrogen, the inhibition to Vibrio spp., and nutrient composition.

**Comments**

This is an interesting manuscript that reports a marine red yeast, *Rhodosporidiums sphaerocarpum* YLY01, which could be used as a potential versatile probiotic in aquaculture. In this study, the authors isolated and purified a marine red yeast strain YLY01 from farming wastewater, which is suggested as a member of *Rhodosporidiums sphaerocarpum*. Through various physiological experiments, the strain YLY01 was found with the abilities to remove ammonia nitrogen in the farming water, inhibit the growth of Vibrio cells and protect the shrimps in normal culture conditions. Based on these interesting findings, the authors suggest that the red yeast strain YLY01 could be developed as a potential aquatic probiotic as well as an effective microbial agent for high-ammonia sewage treatment.

The authors have presented a compelling story with well controlled experiments and clear presentation of the data. The data of the biosecurity of the strain YLY01 to the shrimps would be more complete if the survival rate of the shrimps is tested when incubated with *Vibrio harveyi* and the red yeast strain YLY01 together. Please find the questions and suggestions listed below.

**Major questions and suggestions:**

1. Line 183: The yeast YLY01 has a strong ability to remove ammonia nitrogen in water when little carbon source was added. What about the ammonia nitrogen removal ability with large amount of carbon source? Did the authors test it?

2. Line 185: Why the inhibition of the yeast YLY01 to the growth of Vibrio spp was observed in CFU but not in plate suppression experiment? Could the authors please provide some suppositions to explain it?

3. Line 199: In the assessment of biological safety, did the authors test the survival rate of the shrimps when incubated with *Vibrio harveyi* and the red yeast strain YLY01 together? Can the yeast YLY01 protect the shrimps in the present of *Vibrio harveyi*?

**Minor suggestions:**

1. Line 44: ‘Of those methods’ could be ‘Among those methods’.

2. Line 45: ‘Yeasts as one group of probiotics have been mainly used either as fresh baits…’ could be ‘Yeasts, as one group of probiotics, have been mainly used either as fresh baits…’.

3. Line 202: ‘in 7-day culture’ could be ‘after 7-day culture’.

4. Line 224: The authors could add the full name of ‘His and Lys’, namely, ‘Histidine (His) and Lysine (Lys)’.
